# Early-Onset Neonatal Sepsis: Still Room for Improvement in Procalcitonin Diagnostic Accuracy Studies

**DOI:** 10.1097/MD.0000000000001230

**Published:** 2015-07-31

**Authors:** Claudio Chiesa, Lucia Pacifico, John F. Osborn, Enea Bonci, Nora Hofer, Bernhard Resch

**Affiliations:** From the Institute of Translational Pharmacology, National Research Council (CC), Department of Pediatrics and Child Neuropsychiatry (LP), Department of Public Health and Infectious Diseases (JFO), Department of Experimental Medicine, Sapienza University of Rome, Rome, Italy (EB); and Research Unit for Neonatal Infectious Diseases and Epidemiology, Division of Neonatology, Department of Pediatrics and Adolescent Medicine, Medical University of Graz, Graz, Austria (NH, BR).

## Abstract

To perform a systematic review assessing accuracy and completeness of diagnostic studies of procalcitonin (PCT) for early-onset neonatal sepsis (EONS) using the Standards for Reporting of Diagnostic Accuracy (STARD) initiative.

EONS, diagnosed during the first 3 days of life, remains a common and serious problem. Increased PCT is a potentially useful diagnostic marker of EONS, but reports in the literature are contradictory. There are several possible explanations for the divergent results including the quality of studies reporting the clinical usefulness of PCT in ruling in or ruling out EONS.

We systematically reviewed PubMed, Scopus, and the Cochrane Library databases up to October 1, 2014. Studies were eligible for inclusion in our review if they provided measures of PCT accuracy for diagnosing EONS. A data extraction form based on the STARD checklist and adapted for neonates with EONS was used to appraise the quality of the reporting of included studies.

We found 18 articles (1998–2014) fulfilling our eligibility criteria which were included in the final analysis. Overall, the results of our analysis showed that the quality of studies reporting diagnostic accuracy of PCT for EONS was suboptimal leaving ample room for improvement. Information on key elements of design, analysis, and interpretation of test accuracy were frequently missing.

Authors should be aware of the STARD criteria before starting a study in this field. We welcome stricter adherence to this guideline. Well-reported studies with appropriate designs will provide more reliable information to guide decisions on the use and interpretations of PCT test results in the management of neonates with EONS.

## INTRODUCTION

Early-onset neonatal sepsis (EONS), diagnosed during the first 3 days of life, is a leading cause of morbidity and mortality among infants.^1,2^ Currently, criteria for EONS usually include documentation of infection in a newborn infant with a serious systemic illness in which noninfectious explanations for the abnormal pathophysiologic state are excluded or unlikely.^3^ However, even culture is not free from error because it can be falsely sterile, as suggested by postmortem cultures,^4^ or because of the low yield caused by insufficient sample volumes, intermittent, or low-density bacteremia.^5^ Antibiotic treatment prior to blood culture may further reduce the diagnostic performance of blood culture.^5^ Yet, clinical signs may be minimal and are similar to those of various noninfectious processes. Hence, antibiotics are often started empirically in infants with perinatal risk factors or clinical signs suggestive of bacterial infection.

The availability of a laboratory test to accurately and more rapidly identify septic neonates than is done by the isolation of microorganisms from body fluid specimens would be of considerable value in improving the outcome of this challenging clinical problem, and in minimizing unnecessary treatment of uninfected patients during the immediate postnatal period. Many laboratory tests including various leukocyte indices and acute-phase proteins have been recommended for the evaluation of suspected infection in the neonate.^3^ However, the inability of any single laboratory test to provide rapid, reliable, and early identification of infected (and, as importantly, noninfected) neonates has led to a search for other diagnostic markers.^3^ Among those evaluated in recent years has been procalcitonin (PCT).^6^

In 1994, Dandona et al^7^ showed that after the injection of endotoxins in normal human volunteers, the PCT concentration was undetectable at 0, 1, and 2 hours, but was detectable at 4 hours peaking at 6 hours, and maintaining a plateau through 8 and 24 hours. Because of this rapid response, the body of literature investigating PCT in adults and children with sepsis, as an attractive alternative to C reactive protein (CRP), has grown rapidly over the last 2 decades,^6,8,9^ providing insight as well as posing questions regarding the potential use of PCT for the diagnosis of sepsis. However, clinicians have been less familiar with the potential use of PCT measurements for clinical purposes in neonatal patients,^3^ in particular those presenting EONS.^6^ Increased PCT is a potentially useful diagnostic marker of EONS, but reports in the literature are contradictory.^6^

In contrast to CRP, reports in the literature have shown that PCT is an early and specific marker of neonatal sepsis, confirming the importance of the latter in excluding infection shortly after birth.^10^ However, falsely increased PCT concentrations have been reported for critically ill newborns presenting with “apparently” noninfectious conditions.^11–16^ Although these studies argued for the lack of PCT specificity for the diagnosis of sepsis in neonates, their conclusions should be interpreted with caution. First, arbitrary cut-offs were used to differentiate infectious and noninfectious clinical conditions. For instance, in the report by Lapillonne et al,^16^ uninfected neonates (mean postnatal age, 2.3 days) were deemed to have high serum PCT concentrations on the basis of a surrogate cut-off level originally established in children admitted to a pediatric intensive care unit. Second, interpretation on the use of PCT was complicated by diverse study populations.^10^ Heterogeneity not only within the study group, but also within categories defined as “sepsis,” “distress,” “infected,” “respiratory distress,” or even “hemodynamic failure,” has been huge.^10^ Third, the PCT response was assessed in neonates with wide-ranging differences in postnatal age (hours to weeks),^10^ without consideration of the gestational age and birth weight of the baby.^3^ However, failure to recognize gestational- and age-specific cut-off values over the first few days of life may confound the interpretation of what constitutes a negative and a positive PCT value for the diagnosis of EONS.^17–21^ Fourth, PCT levels obtained from uninfected patients were not compared with PCT reference values for each time point of evaluation. This limitation makes it difficult to determine which neonatal factors may cause a significant deviation. Fifth, it is uncertain how the infectious state in the “uninfected” neonates was ruled out. In the report by Monneret et al,^11^ high PCT concentrations were reported during the first 4 days of life in apparently “uninfected” newborns who presented with respiratory distress syndrome (RDS) of various etiologies. However, it would be of interest to know how many patients with severe RDS demonstrated clinical evidence of being “uninfected.” Finally, absent from these studies are data on how the interpretation of the PCT response in the uninfected as well as in the infected neonates might have been hampered by the severity of the underlying illnesses (and their extent of inflammatory reaction).^6,22^ It is clear from the above that the evaluation of the clinical usefulness of PCT in ruling in or ruling out neonatal sepsis, in particular EONS, is dependent on study consistency. In this article, we therefore performed a systematic review assessing accuracy and completeness of PCT diagnostic studies for EONS including key elements of design, conduct, and analysis according to the Standards for Reporting of Diagnostic Accuracy (STARD) statement.^23,24^

## METHODS

Our systematic review was conducted, when possible, in accordance with the Preferred Reporting Items for Systematic Reviews and Meta-Analyses (PRISMA) guidelines. Ethical approval was not necessary for this review study.

### Data Sources

We systematically reviewed PubMed, Scopus, and the Cochrane Library databases up to October 1, 2014. The PubMed combined search term used was: (Procalcitonin OR PCT) AND (neonatal sepsis OR neonatal infections OR neonate). The search terms applied to the Scopus and the Cochrane Library were “Procalcitonin and neonate” and “Procalcitonin,” respectively. The bibliographies of relevant articles were also hand-searched.

### Study Selection Criteria

Studies were eligible for inclusion in our review if they provided measures of PCT accuracy for diagnosing EONS, defined by the National Institute of Child Health and Human Development and Vermont Oxford Networks as sepsis with onset at ≤3 days of age.^25^ We excluded studies that used a PCT semiquantitative assay. Duplicate articles, conference abstracts, or studies written in languages other than English were also excluded.

### Data Extraction

Data extraction was performed independently by 2 authors and included the country of the research, year of publication, journal, reference standard employed, the type of the study design, the number and specific characteristics of the patients in the septic and nonseptic groups (Table 1  ), and items related to the quality of the methods and reporting (listed below). Specific data regarding the PCT cut-off level used, the sensitivity, specificity, and area under the receiver operating characteristic (ROC) curve for the diagnosis of EONS were also extracted (Table 1  ). In cases in which estimates of uncertainties around the observed values of sensitivities and specificities were not calculated using Wilson method,^42^ we calculated them.

**TABLE 1:**
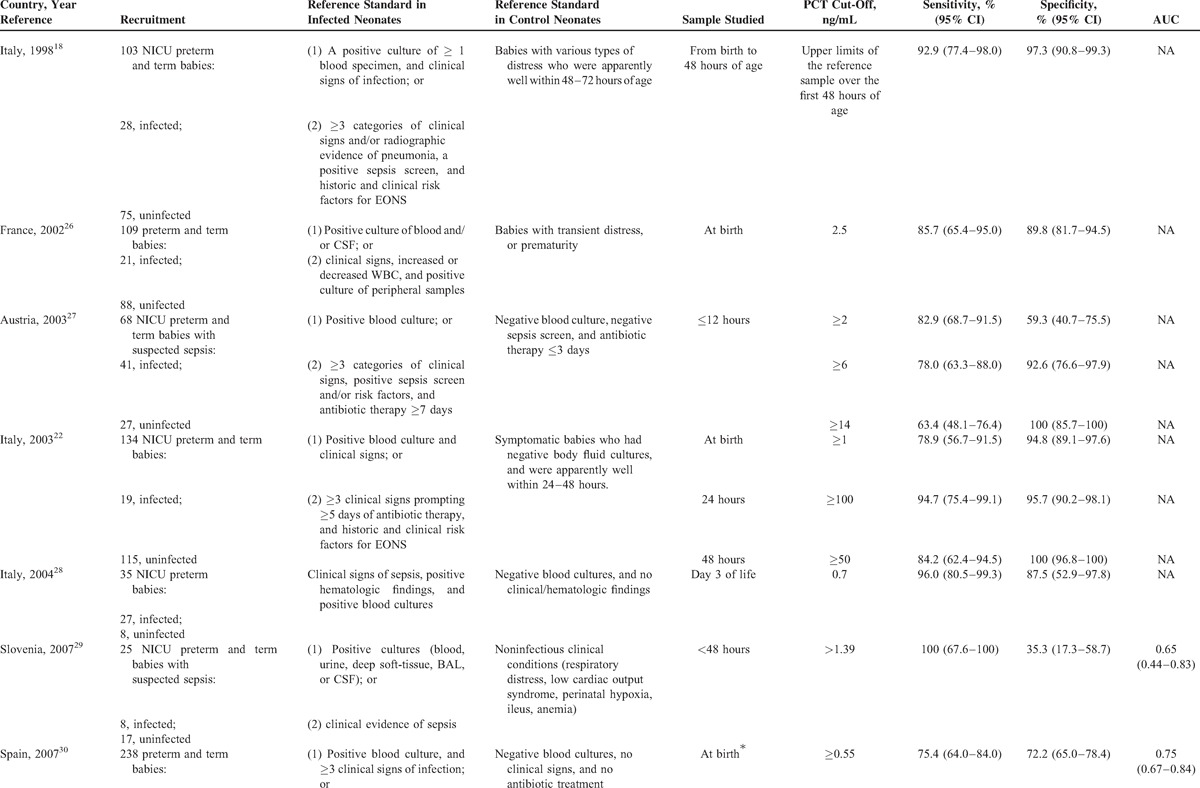
Characteristics of PCT Accuracy Studies (1998–2014) for Diagnosing Early (≤72 hours)-Onset Neonatal Infection

**TABLE 1 (Continued):**
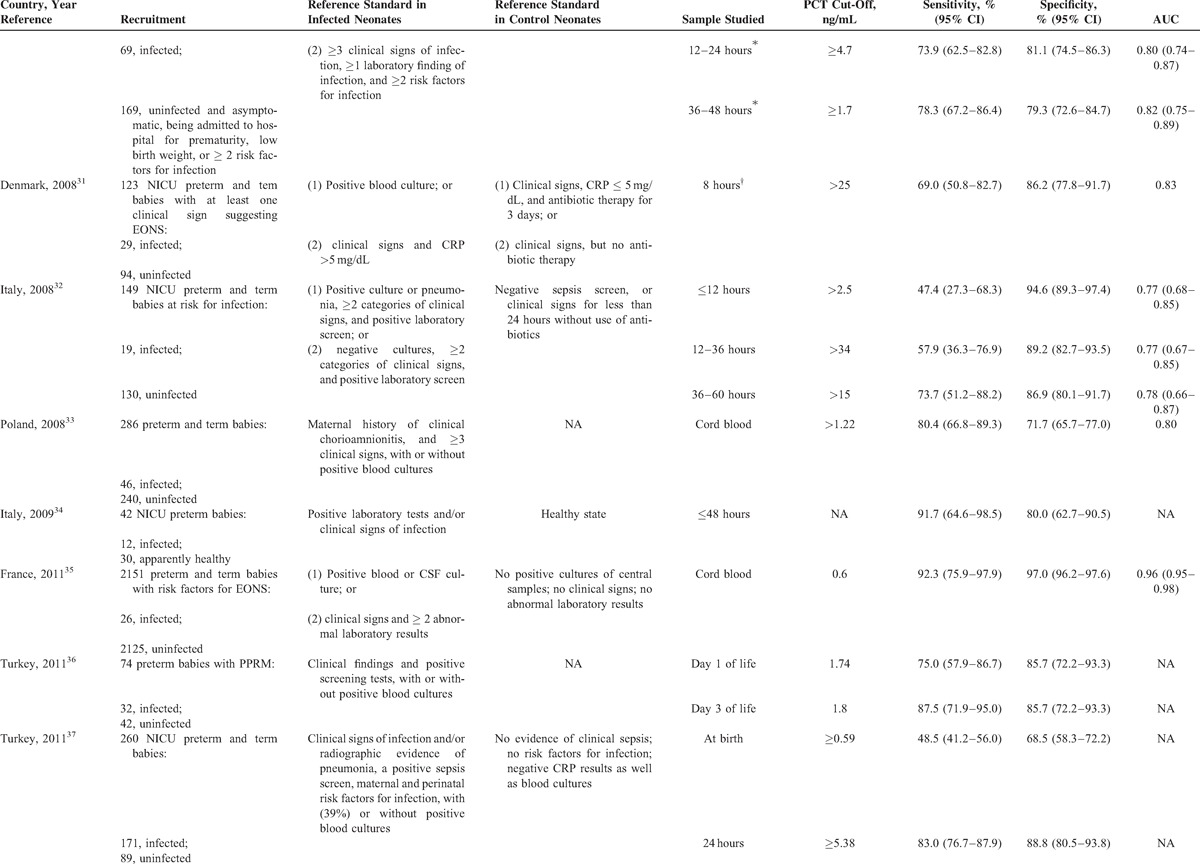
Characteristics of PCT Accuracy Studies (1998–2014) for Diagnosing Early (≤72 hours)-Onset Neonatal Infection

**TABLE 1 (Continued):**
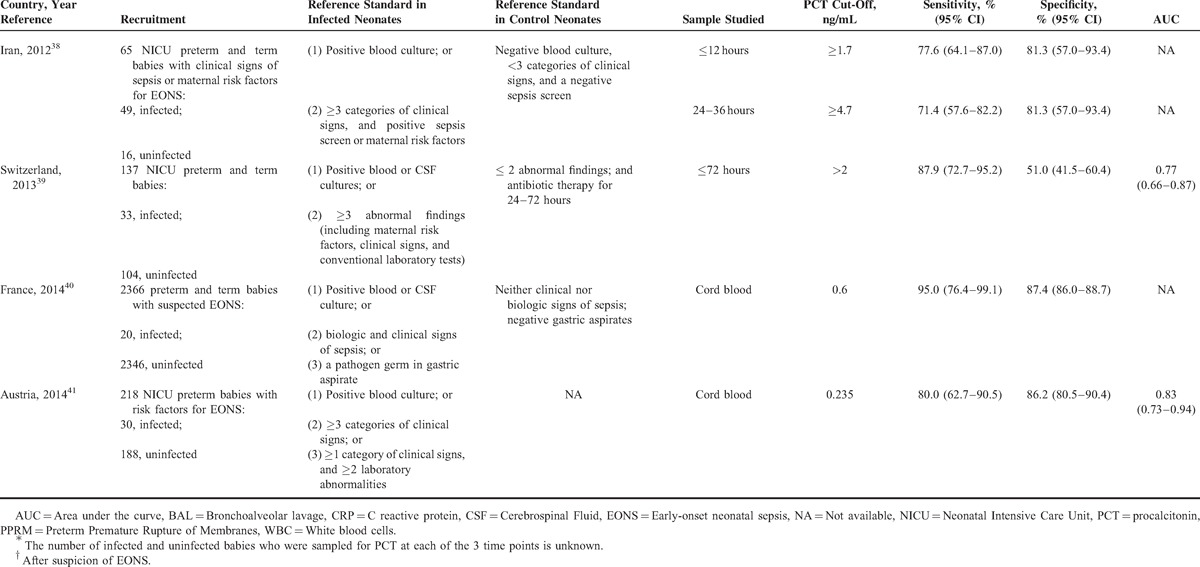
Characteristics of PCT Accuracy Studies (1998–2014) for Diagnosing Early (≤72 hours)-Onset Neonatal Infection

### Quality Assessment of the Included Studies

In 2003, the STARD statement was published in 13 biomedical journals.^23,24,43^ The STARD initiative was developed in response to accumulating evidence of poor methodological quality. Therefore, a data extraction form based on the STARD checklist and adapted for neonates with EONS was used to appraise the overall quality of the included studies which is presented in Table 2. All articles were closely examined to gauge the extent to which they adhered to the STARD checklist by assigning a yes or no response to each item.

**TABLE 2:**
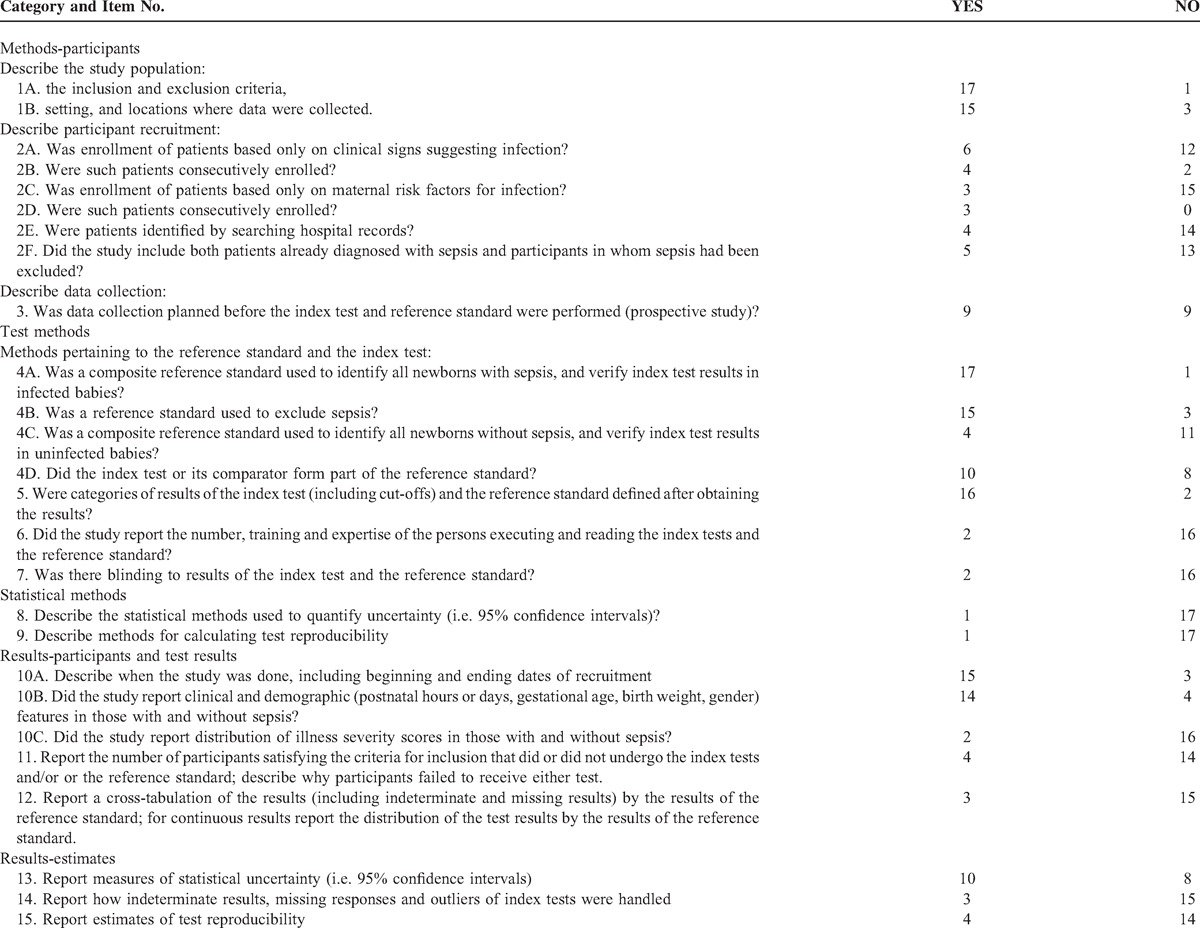
Quality of Reporting of PCT Accuracy Studies (1998–2014) for Diagnosing Early (≤72 hours)-Onset Neonatal Infection

Twenty-five items makeup the STARD checklist. When the papers considered in this study were assessed, only those STARD items that have been empirically shown to have a potentially biasing effect on the results of diagnostic accuracy studies^44–47^ and those items we deemed they may account for variation between studies in estimates of diagnostic accuracy for neonatal sepsis were evaluated. The following features, with corresponding STARD checklist item number, appear to have a possible biasing effect^44–47^ or account for variation between diagnostic studies on neonatal sepsis: a description of the target population (STARD: item #3); a description of where (primary, secondary, or tertiary care setting) patients were recruited and where the test and the reference standard were performed (STARD: item #3); a description of how eligible subjects were recruited (ie, a description whether the study included: neonates who presented only symptoms suggesting sepsis; neonates who presented only maternal risk factors for infection; neonates in whom diagnosis of sepsis had been already established or excluded; a neonatal population selected from existing data files [STARD: item #4]); a description of whether recruitment involved either a consecutive series of patients with and without sepsis or a subselection (STARD: item #5); a description of the data collection process (STARD: item #6); a description of the reference standard for diagnosis (or exclusion) of neonatal sepsis and its rationale (ie, a description of whether: a single or composite reference standard [reflecting 2 or more reference standards] was used to identify all newborns positive to the test for sepsis and verify the index test results in infected babies; a reference standard [along with pertinent details: a single or composite reference standard reflecting 2 or more reference standards or noninfectious clinical conditions-STARD: item #18] was used to exclude EONS and to verify the index test results in uninfected babies; the reference standard was independent of the index test [ie, whether the index test or its comparator formed part of the reference standard in neonates with or without sepsis—STARD: item #7]); a description of whether categories of results of the index test (including cut-offs) and the reference standard were defined before or after obtaining the results (STARD: item # 9); information concerning the number and training of the persons executing and evaluating the index test and the reference standard (STARD: item #10); a description of whether or not the readers of the index tests and the reference standard were blind (masked) to the results of the other test (STARD: item #11); a description of the methods for calculating the precision around used measures of diagnostic accuracy (STARD: item #12), and the reporting of the range within which the true values were likely to lie (STARD: item #21); a description of the methods used for calculating test reproducibility (STARD: item #13), and the reporting of the estimates of test reproducibility (STARD: item #24); a description of the study population including septic and nonseptic neonates in which tests were executed (study period, clinical and demographic features, distribution of illness severity scores [STARD: items #14, #15, and #18]); a description of how many participants fulfilling inclusion criteria failed to undergo the index test or reference standard and the reasons of failing to do so (STARD: item #16); a cross-tabulation of the results of the index test by the results of the reference standard, and for continuous results, a description of the distribution of the test results by the results of the reference standard (STARD: item #19); and a description of how indeterminate results, missing data, and outliers of the index test were handled (STARD: item #22).

Adequate reporting of 3 key domains, that is, descriptions of participant recruitment, reference standard and index test, and study population, was considered essential for capturing the global and integrative quality of reporting the accuracy of diagnostic tests for ruling in or ruling out EONS, and therefore they were split into various complementary items.

Two investigators (CC, BR) independently assessed the methodological quality of all eligible studies. The interrater agreement between these 2 reviewers was expressed as overall agreement percentage and more formally tested with the kappa statistic.

## RESULTS

### Study Selection

**FIGURE 1:**
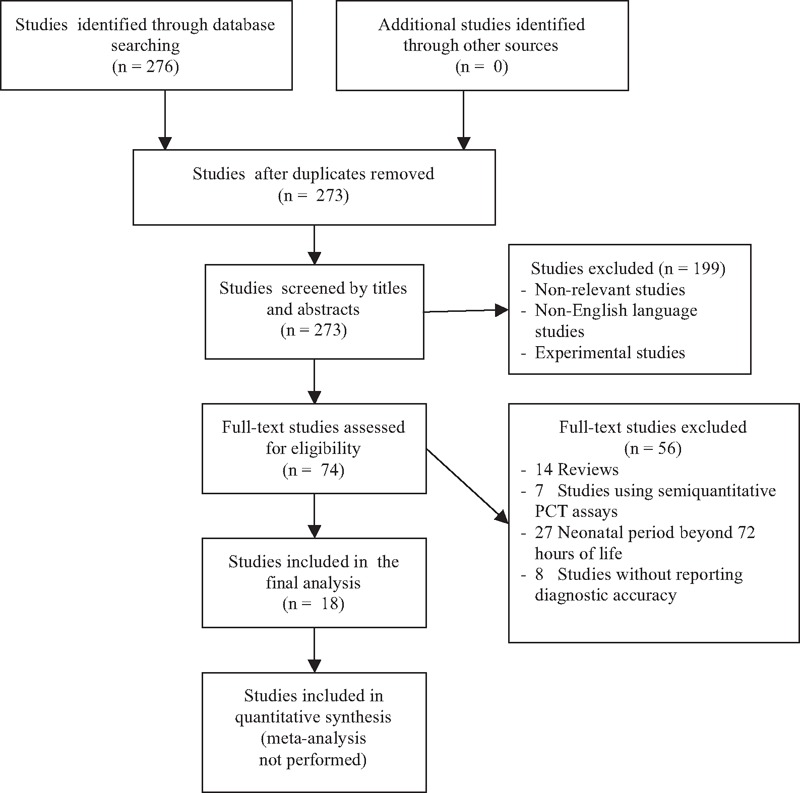
Schematic flow chart for the selection of studies.

In Figure 1, we present the flow diagram as recommended by the PRISMA statement showing the process of the selection of the studies included in our review. Specifically, our search identified a total of 273 articles (after removing the duplicates). According to titles and abstracts, 74 of them were selected for further assessment. After a review of full-text, 18 were eligible for inclusion in our review.^18,22,26–41^ All articles were identified as studies of diagnostic accuracy and stated this research objective in the introduction. Papers were published between 1998 and 2014. Most studies (15/18) were performed in single perinatal centers.

### Characteristics of the Included Studies

In Table 1  , we present the main characteristics of the 18 included studies. Half of the studies had a retrospective design, while the remaining were prospective. Fourteen studies included both preterm and term infants and the remaining 4 involved only preterm newborns. The total number of patients included in the 18 studies was 6547, of whom 680 had EONS and 5867 were uninfected. In all studies, the septic group consisted of neonates with culture proven and/or clinically diagnosed EONS. The nonseptic group consisted of ill neonates with other conditions that were hospitalized in the neonatal unit in all but one of these studies. There was considerable variation in the reference standard used for diagnosing (or excluding) EONS and thus verifying index test results.

### Diagnostic Accuracy of the Included Studies

Table 1   summarizes PCT diagnostic thresholds and accuracy measures such as PCT sensitivities and specificities for identifying (or excluding) EONS. There was over the first 72 hours of life wide variation in PCT sampling times, PCT cut-off values, and results in PCT diagnostic accuracy. The minimum and maximum PCT cut-off used was 0.235 and 100 ng/mL, respectively. The observed values of sensitivity ranged from 47.4% (95% confidence interval [CI] 27.3–68.3) to 100% (95% CI 67.6–100) and specificity from 35.3% (95% CI 17.3–58.7) to 100% (95% CI 96.8–100).

### Quality of Reporting of PCT Diagnostic Studies

We evaluated the 18 included studies for compliance with STARD guidelines. Agreement between the 2 reviewers was very good with an overall agreement percentage of 96%. The kappa statistic had a value of 0.93 (95% CI 0.91–0.95), indicating a very good agreement. Discrepancies were rectified by consensus.

Table 2 summarizes the extent to which the 18 studies adhered to the STARD items. The reporting of individual items showed wide variation. Overall, the quality of reporting of PCT diagnostic accuracy studies on EONS over the last 2 decades was suboptimal. Information on key elements of design, conduct analysis, and interpretation of test accuracy were frequently missing.

## DISCUSSION

Studies of the PCT accuracy for the diagnosis of EONS have produced discrepant results in the past. Potential sources of the wide variation in PCT sensitivity and specificity include the lack of consistent PCT cut-off values regardless of the time of sampling, and differences in study sample sizes, patient demographic and clinical characteristics, and participant recruitment.

We therefore evaluated the quality of reporting of PCT accuracy studies for diagnosing EONS. The importance of describing how eligible subjects were identified cannot be overemphasized. Selection of a study population is critical in appraising a diagnostic test.^48^ It is crucial to describe the population from which patients and patient controls originated, as it allows an assessment of the population “spectrum of disease.” Reported estimates of diagnostic accuracy may have limited clinical applicability in external populations (generalizability) if the spectrum of tested patients is not similar to the patients for whom the test will be used in practice.^49,50^ Our analysis showed that while some PCT accuracy studies enrolled only neonates who were suspected of having the disease because of presenting symptoms, other studies recruited neonates who were (initially asymptomatic and) only at risk of developing the disease because of history of maternal risk factors. Yet, other studies included neonates already diagnosed with sepsis and participants in whom sepsis had been already excluded. Finally, there were also designs starting with: 2 separate selection processes to sample patients with sepsis and patients without sepsis (a sampling method producing biased estimates of accuracy known as “the diagnostic case–control” with limited spectrum);^44,51^ nonconsecutive sampling of patients (a sampling method leading to the “limited challenge bias”);^52^ retrospective data collection; and identification of patients by searching hospital records. These alternative study designs are likely to influence the spectrum of disease in the included patients, and estimates of diagnostic accuracy. In a study of evidence of bias and variation in diagnostic accuracy studies, Rutjes et al^46^ found that the largest overestimation of accuracy was found in studies that included severe cases and healthy controls and in those in which 2 or more reference standards were used to verify index test results. The design features associated with overestimation of diagnostic accuracy were also nonconsecutive inclusion of patients, and retrospective data collection.^46^ In contrast, the selection of patients on the basis of whether they had been referred for the index test or on the basis of previous test results, rather than on clinical symptoms, was significantly associated with lower estimates of accuracy.^46^

The spectrum of sepsis as well as the range of other conditions that occur in patients suspected of sepsis can vary from setting to setting, depending on what referral mechanisms were in play.^53–55^ Thus, the accuracy of tests may vary between primary care and secondary or tertiary care.^24^ Most PCT accuracy studies on EONS reported this issue properly by specifying that the neonatal intensive care setting was the healthcare setting where data were collected.

Spectrum bias also results from differences in the severity of sepsis/EONS between populations. It may be that some of the variation in PCT sensitivity in the diagnosis of EONS among published reports might have reflected differences in baseline severity and risk status. Although individual clinical and demographic attributes of the septic and nonseptic neonatal populations were reported in most PCT diagnostic accuracy studies (14/18), proxy measures of morbidity such as gestational age and birth weight do not capture the overall morbidity status. Therefore, it is of greater concern that reporting of the distribution of scores of major measures of illness severity was poor in neonates with and without sepsis.

Differential verification bias is also a key issue in any diagnostic accuracy study. It occurs when some of the index test results are verified by one type of reference standard and other results by a different standard.^49^ The effect of differential verification depends on the quality of the different reference tests used. Differential verification poses a problem if the reference standards differ in accuracy and if the choice of reference standard relates to the results of the index test.^49^ This usually occurs when patients testing positive on the index test receive a more accurate reference standard than those testing negative. Previous studies that relied on 2 or more reference standards to verify the results of the index test reported estimates of diagnostic accuracy on average 60% higher than those encountered in studies that used a single reference standard.^46^ The origin of this difference probably resides in differences between the reference standards in how they define sepsis, or in their quality.^50^ Reference standards are not interchangeable. They may not have the same degree of error and may not identify the same segment of the disease spectrum. Thus, worrisome is the fact that in most of the included PCT diagnostic accuracy studies different reference standards were used to diagnose (or exclude) EONS and verify index test results.

A further step in the critical appraisal of PCT accuracy studies on EONS is whether the reference standard was independent of the index test and the comparator of the index test.^48,56,57^ When the result of the index test or its comparator is used in establishing the reference standard, incorporation bias may occur. Unfortunately, in 10 (55.5%) of the 18 included studies, the comparator of the index test such as CRP test was also a component of the reference standard. In such situations, it is likely that the person interpreting the results of the comparator will have knowledge of the results of the other test (index test and reference standard).^50^

In order to make a valid comparison between the index test and the standard test, it is essential that the criteria (cut-off values, etc.) are defined before the start of the study. If the cut-offs are decided after the results are obtained, the likelihood that another study will replicate the findings is reduced. Apparently most studies (16/18) included cut-offs post hoc.

We also determined whether the interpretation of the index test or reference standard was influenced by knowledge of the results of the other test. Interpretation of the results of the index test may be influenced by knowledge of the results of the reference standard, and vice versa.^48,50^ This may lead to inflated measures of diagnostic accuracy. Information about masking was reported in only 2 of the 18 evaluated reports.

Methods for calculating PCT test reproducibility and measures of test reproducibility were reported poorly, by only 1 and 4 of the 18 included articles, respectively. There may be a lack of understanding about the implications of poor reproducibility on the overall utility of a test. Furthermore, the value of sensitivity and specificity estimates are unclear in the absence of precise information about test reproducibility.

Information regarding the number, training, and expertise of the persons involved in the execution, and reading of the index test and the reference standard was also among the least commonly reported items of the STARD guidelines. There may be a lack of understanding of the effects of the low level of expertise on the final outcome of the diagnostic accuracy of a test. On the other hand, knowledge of the numbers, training, and experience of operators would help to estimate the repeatability of the test results in different settings.

Measures of diagnostic accuracy will be biased if the result of the index test influences the decision to order the reference standard (an effect known as “partial verification bias”).^48,58–64^ According to the STARD statement,^24^ partial verification bias occurs in up to 26% of diagnostic studies.^61^ It is therefore important to describe how many participants satisfying inclusion criteria failed to undergo index or reference tests and the reasons of failing to do so. This item was reported in a minority of the included publications (4/18). A flow diagram is highly recommended to illustrate the design of the study and provide the exact number of participants at each stage of the study;^23,24^ only 2 of the 18 PCT diagnostic accuracy studies had a flow diagram.

Since the reported values of sensitivity, specificity, and area under the ROC curve are only estimates, measures of uncertainty (such as CIs) give readers a range within which true values may lie and indicate the precision of the diagnostic test.^65^ CIs were reported in more than half (11/18) of the included studies. However, only one of the 18 studies adequately reported and cited the statistical methods used to quantify these estimates of diagnostic accuracy.

Diagnostic tests may report uninterpretable results for some patients, or call results uncertain, indeterminate, or intermediate. The frequency of such results can vary widely between tests.^57^ These problems are sometimes not reported in diagnostic accuracy studies or ignored in analyses.^66^ The frequency of these results, by itself, is an important clue of the overall utility of a test.^67,68^ This item was reported in only 3 of the 18 studies.

Since the technology for existing tests is rapidly improving, it is important to report the actual dates when the study was performed. This will allow the reader to consider any technologic advances since the study was done. Fortunately, this information was provided in most publications.

In general, the results of our analysis of PCT diagnostic accuracy studies for EONS provide further evidence of the importance of design features in studies of diagnostic accuracy. Studies of the same test can produce different estimates of diagnostic accuracy depending on choices in design. We feel that our results should be taken into account by researchers when designing new PCT accuracy studies for diagnosis of EONS as well as by reviewers and readers who appraise these studies. In that vein, initiatives such as STARD should be endorsed to improve the awareness of design features, the quality of reporting and, ultimately, the quality of study designs before starting a PCT study.

## CONCLUSIONS

With respect to the clinical usefulness of PCT in diagnosing or ruling out EONS, it is time to debate the methods used to measure PCT performance, rather than just how a given test performs. To interpret correctly the results of PCT accuracy studies for EONS, readers must understand the design, conduct, and data analysis and must be able to judge the internal validity and generalizability of the results. This goal can only be achieved through complete transparency of the reporting of the articles. The results of our analysis involving PCT accuracy in diagnosing EONS show that the quality of reporting for many of the STARD items, that have been shown to have a potentially biasing effect on the results of diagnostic accuracy studies or appear to account for variation between studies, is substandard. For this reason, this article does not include any summary results or meta-analysis. Authors and peer reviewers are encouraged to adhere to and enforce the STARD guidelines, because there is clearly room for improvement in the reporting of PCT diagnostic accuracy studies for EONS.
